# Virtual left atrial appendage occlusion in paroxysmal atrial fibrillation during sinus rhythm predicts variable reductions in blood stasis

**DOI:** 10.1113/JP288587

**Published:** 2025-07-10

**Authors:** Sophia Bäck, Jonas Lantz, Iulia Skoda, Lars O. Karlsson, Anders Persson, Carl‐Johan Carlhäll, Tino Ebbers

**Affiliations:** ^1^ Department of Health, Medicine and Caring Sciences Linköping University Linköping Sweden; ^2^ Center for Medical Image Science and Visualization Linköping University Linköping Sweden; ^3^ Department of Cardiology in Linköping Linköping University Linköping Sweden; ^4^ Department of Radiology in Linköping Linköping University Linköping Sweden; ^5^ Department of Clinical Physiology in Linköping Linköping University Linköping Sweden

**Keywords:** atrial fibrillation, computational fluid dynamics (CFD), flow component analysis, left atrial appendage occlusion

## Abstract

**Abstract:**

Left atrial appendage occlusion (LAAO) is an emerging treatment option for cardioembolic stroke risk reduction in patients with sustained or paroxysmal atrial fibrillation (AF). How LAAO affects the atrial blood flow field during sinus rhythm has not yet been defined. Here, we simulated virtual LAAO in 21 paroxysmal AF patients and eight controls in normal sinus rhythm using computational fluid dynamics (CFD). We found that in AF patients, LAAO reduces the amount of blood that remains in the LA for more than three cardiac cycles to levels similar to the control group with intact LAA. However, there is a large variation among the AF group and even after LAAO the patients with highest stasis still had a relatively large amount of blood remaining in the LA over multiple cycles. This remaining blood is predominately located close to the site of LAA occlusion, a region where occlusion device related thrombi are known to occur. Accurate patient stratification is important to identify the impacts of LAAO on the patient specific haemodynamics.

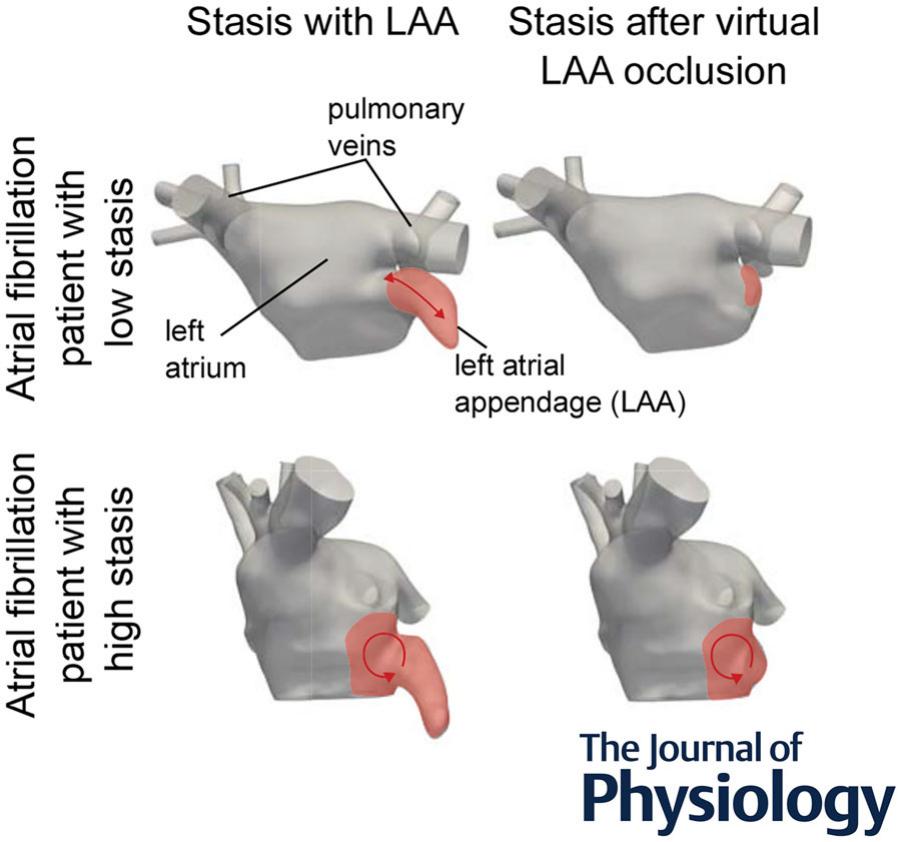

**Key points:**

- Patients with atrial fibrillation (AF) have an increased risk for stroke. One underlying mechanism for this is that thrombi form in the left atrial appendage (LAA).
- To reduce the risk of thrombi forming in the LAA, it can be closed with an occlusion device.
- In this study, we simulated how the blood flows in the left atrium of AF patients before and after a virtual LAA occlusion and compared it to a control group.
- We found that LAA occlusion could reduce the time blood stays in the left atrium in most patients with AF to similar levels as the control group. But in some patients, blood stagnated for a long time even after LAA occlusion.
- Our results help us understand why thrombi can form even after LAA occlusion and might help to predict which patients could benefit most from LAA occlusion.

## Graphical abstract

**Abstract figure legend** Stasis before and after left atrial appendage occlusion (LAAO) in participants with high stasis and low stasis. Stasis is predominantly located in LAA and reduced after LAAO. But in the participant with high stasis, stasis remains close to the occlusion site, indicating a potential risk for device related thrombi.

## Introduction

Patients with atrial fibrillation (AF) can experience symptoms due to fast heart rate as well as heart irregularity, and AF can be responsible for the development of heart failure. These factors may require in some instances life‐style management, medication or catheter‐based interventions in order to restore normal sinus rhythm. Furthermore, AF is associated with an increased risk of stroke, mainly due to thrombi that form in the left atrial appendage (LAA). Currently, the main treatment strategy to reduce stroke risk is the prescription of oral anticoagulants (OAC), especially direct oral anticoagulants (DOAC), which have fewer bleeding complications compared to treatments such as warfarin (Joglar et al., 2024). Alternatively, LAA occlusion (LAAO) or LAA removal is being investigated (Alkhouli et al., 2022), where the LAA is either percutaneously occluded with an occlusion device or surgically removed. LAAO alters the atrial haemodynamics by removing the region with the most blood stasis. While this treatment has shown to be a promising alternative to oral anticoagulation (Osmancik et al., 2020; Reddy et al., 2017), current clinical guidelines are waiting for stronger evidence for LAAO, especially in patients who are not suitable for OAC (Hindricks et al., 2020; Joglar et al., 2024). It is unclear whether patients in the early stages of AF could benefit from LAAO, or how the LAAO affects left atrial haemodynamics in these patients.

While LAAO reduces the risk for stroke similarly to OAC (Osmancik et al., 2022), up to 5% of LAAO patients develop LAAO device‐related thrombi (DRT). DRT is associated with a more than three times increased risk for ischaemic stroke (Alkhouli et al., 2022). A large randomized controlled trial found that occlusion or surgical removal of the LAA reduces ischaemic strokes from 6.9 to 4.6% (Whitlock et al., 2021). For percutaneous LAAO, some risk factors for DRT have been identified, but the patient‐specific risk stratification remains challenging (Alkhouli et al., 2022).

An important factor in thrombus formation is blood stasis. Regions of low flow velocities in the left atrium are difficult to measure directly. Echocardiography usually only provides two dimensional images and limited flow definition, and 4D flow MRI has a relatively low spatial resolution and the data is acquired over many cardiac cycles, which hampers the data quality in atrial arrythmia. Alternatively, the atrial blood flow can be calculated based on time‐resolved CT images using computational fluid dynamics (CFD), and this allows regions of low velocities to be quantified (Bäck, Skoda et al., 2023). This approach has demonstrated velocity patterns and flow rates comparable to *in vivo* 4D flow MRI (Bäck, Henriksson et al., 2023; Lantz et al., 2018). Various studies have used CFD to study the effect of LAAO compared to the LA with intact LAA (Enomoto et al., 2024; Ghodrati‐Misek et al., 2022; Jia et al., 2019), different occlusion procedures (Aguado et al., 2019; D'Alessandro et al., 2023, 2020; Zhong et al., 2024) and the occurrence of DRT (Albors et al., 2024; Mill et al., 2021; Planas et al., 2022; Vogl et al., 2024). They have shown that CFD can be helpful in LAAO treatment planning, but most studies were limited to small numbers of patients. Two recent studies, however, investigated the occurrence of DRT in relatively large patient groups (Albors et al., 2024; Vogl et al., 2024). Vogl et al. found larger atrial volume and lower wall shear stress on the top surface of the device in patients developing DRT (Vogl et al., 2024). These studies were performed on patients planned for LAAO, who typically have severe symptoms and atrial dysfunction. The effects of LAAO on patients in the early stages of AF have not been investigated yet.

The aim of the current study is to evaluate how LAAO impacts the atrial haemodynamics of paroxysmal AF patients during sinus rhythm. These patients are early in the AF disease and currently not considered for LAAO in routine clinical practice. We perform our analysis by simulating the atrial haemodynamics and estimating atrial stasis in patients both with an intact LAA and following virtual LAA occlusion. This information could increase our understanding of the effect of LAAO on atrial haemodynamics and help identify new patient groups that could benefit from LAAO, as well as provide patient‐specific risk stratification.

## Methods

### Ethical approval

All study participants provided informed written consent for the study. The study conforms to the standards set by the 2024 version of the *Declaration of Helsinki* except for registration in a database. The study has been approved by the local ethics board (Regionala etikprövningsnämnden i Linköping, 2018/275‐31 and 2017/502‐31).

### Patient characteristics and image acquisition

In this study, patients with paroxysmal AF were compared to a control group without a history of AF, whom were all recruited at Linköping University Hospital. The individuals with paroxysmal AF had a clinical referral for time‐resolved cardiac CT before catheter ablation and the CT was acquired in sinus rhythm. Patients with ongoing AF on the date of examination, previous cardiac surgery or ablation, more than moderate mitral valve regurgitation, more than moderate left ventricular dysfunction or dilatation, or uncontrolled hypertension were excluded from the study. Uncontrolled hypertension was an exclusion criterion since high pressure in the aorta would affect the haemodynamics in the entire left heart. One AF patient was excluded from the study, due to poor image quality, leaving 21 participants in this group. The control group were patients who had a clinical referral for coronary CT angiography, and participants with a history of AF and more than mild valvular regurgitation were excluded from the study. In this group, three participants were removed because part of the LA and LAA were missing in the CT image, and one participant was excluded because of AF diagnosis one month after the CT examination, so this group consisted of eight individuals (Table 1).

**Table 1:** Participant characteristics

	Controls (*n* = 8)	AF group (*n* = 21)	*P* value
Age (years)	60 ± 14	67 ± 9	0.15
Women *n* (%)	3 (38)	6 (29)	0.84
Body surface area (m^2^)	2.13 ± 0.28	2.02 ± 0.17	0.22
Height (m)	1.73 ± 0.07	1.78 ± 0.09	0.17
Body mass index	32 ± 6	26 ± 2	**<0.001**
Heart rate	67 ± 7	62 ± 8	0.16
Systolic blood pressure (mmHg)	139 ± 17	145 ± 20	0.47
Diastolic blood pressure (mmHg)	76 ± 15	81 ± 12	0.37

Data are presented as mean± standard deviation. A two‐sample *t* test with a significance level of 5% was used for the inter group comparisons.

The CT images were acquired on a dual‐source CT scanner (Somatom Force, Siemens Healthineers, Erlangen, Germany), using iodinated contrast medium and reconstructed to 20 timeframes, each representing 5% of the RR interval. More scan details can be found in Bäck, Skoda et al. (2023).

### CFD simulations and virtual LAA occlusion

**Figure 1:**
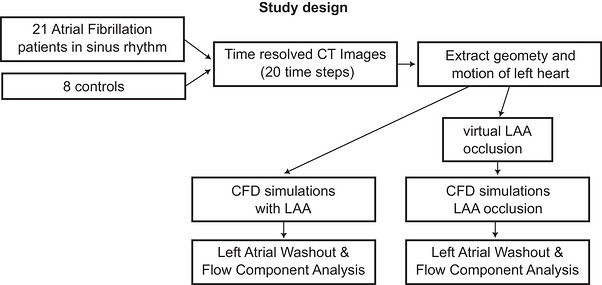
Flow chart of the study design CT: computed tomography; LAA: left atrial appendage; CFD: computational fluid dynamics.

For each participant, two CFD simulations were performed, one with the LAA and one after virtual LAA occlusion. The study design is shown in Fig. 1. The simulations where the LAA was retained were the same as in Bäck, Skoda et al. (2023).

The blood flow in both the left atrium and the left ventricle was simulated. All simulations were performed using Ansys Fluent version 2019 R3. The motion of the endocardium was extracted from the time‐resolved CT images, as described in Bäck, Henriksson et al. (2023). This motion was set as the boundary condition at the walls, while zero relative pressure was prescribed at the pulmonary veins and the ascending aorta. The mitral valve and aortic valve were set to either fully open or closed. The flow rate through the mitral valve was an implicit result of the ventricular motion and the general flow field is driven by the contraction and expansion of the left atrium and left ventricle. This approach has shown good agreement to *in vivo* 4D flow MRI measurements (Lantz et al., 2018). The computational time step was 0.5 ms and the computational meshes contained between 5 and 12 million cells, depending on the size of the heart and the phase of the cardiac cycle. Blood was considered to be a Newtonian fluid with a viscosity of 3.5 × 10^−3^ Pa s. This is a simplification of non‐Newtonian behaviour of blood but has been shown to not affect the rank ordering among subjects (Sanatkhani et al., 2023). Blood density was set to 1060 kg/m^3^ and laminar flow was assumed.

To estimate atrial haemodynamics and stasis, the velocity fields were exported from the CFD simulations at a temporal resolution of 0.025 s, and path lines were created from this exported velocity field of one cardiac cycle starting with a diastolic phase. The flow field of the simulations with LAA was evaluated after simulating five full cardiac cycles, representing a converged velocity field. For the virtual LAAO occlusion, the solution with the LAA after six cardiac cycles was used as an initial condition and the flow field was simulated for another two full cardiac cycles and one systolic phase. The last full cardiac cycle containing a diastolic and systolic phase was analysed, which had a converged velocity field. For the LAA occlusion, the appendage was removed such that the atrial wall was continuously smooth, resembling an optimal occlusion placement or an optimal surgical exclusion. The wall motion of the remaining atrial wall and the left ventricle were kept the same as in the simulation with LAA. This means that the ventricular stroke volume and thus the mitral valve flow were the same in both cases. Due to the non‐existent expansion and contraction of the LAA in the LAAO simulation, the flow through the pulmonary veins was slightly different. More information on the simulation setup can be found in Appendix.

### Stasis assessment

To assess the level of stasis in the left atrium, two different techniques were used: blood washout analysis and atrial flow component analysis. Both of these methods were conducted through particle tracking based on the exported velocity field of one cardiac cycle, which was looped several times. The techniques are described in more detail in the Appendix.

The blood washout was computed by seeding particles in the LA and following them until they leave the LA volume. The blood washout in the LA after 1, 2 and 3 cardiac cycles was calculated from the CFD results using ParaView. For this, the flow fields were exported from CFD simulations every 0.025 s for one cardiac cycle. Particle traces were then looped through this cycle three times, and the timing of particles leaving the left atrium was extracted. Based on this timing, the fraction of blood remaining in the LA after 1, 2 and 3 cardiac cycles could be determined.

**Figure 2:**
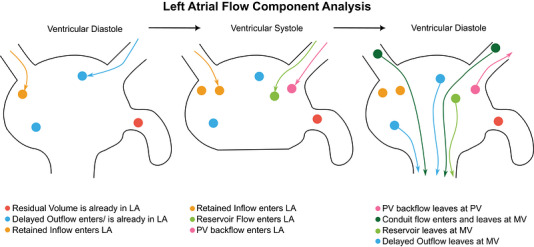
Definition of the flow components Residual volume stays in LA over two heartbeats. Delayed outflow enters before systole and leaves during diastole. Retained inflow enters LA during previous cardiac cycle but does not leave. Reservoir flow enters LA during systole and leaves during diastole. Pulmonary vein (PV) backflow leaves through pulmonary veins. Conduit flow enters and leaves the left atrium (LA) during diastole. Graphic was modified based on Fig. 1 originally published in Bäck et al. (2024) under CC BY 4.0.

For a more detailed analysis of the atrial blood flow, flow component analysis as described Bäck et al. (2024) was also performed. Atrial flow component analysis separates the atrial volume and haemodynamics into six flow components by tracking fluid particles both forwards and backwards in time over two cardiac cycles (Bäck et al., 2024). The particle traces were classified based on their route through the atrium (see Fig. 2). Conduit flow describes the volume of blood that travels through the left atrium from the pulmonary veins to the mitral valve during one ventricular diastolic phase. Reservoir volume is the amount of blood entering the LA during the previous ventricular systole and leaving through the mitral valve during diastole. All blood that has been in the LA for longer than the previous systole and leaves through the mitral valve is classified as delayed outflow. Retained inflow entered the left atrium during the previous cardiac cycle but did not leave in the following heartbeat. Blood that entered the atrium and then returned to the pulmonary veins (PV) is characterized as pulmonary vein backflow. Blood that stayed in the atrium for two consecutive heart beats is defined as residual volume, which here was considered as a marker for stasis. The particle tracking was performed using ParaView version 5.12.

### Statistics

Statistical analysis was performed on Matlab version 2022b. The volumes of the flow components and the remaining blood fraction in the washout analysis before and after virtual LAAO were compared on an individual level using the Wilcoxon signed rank test (paired test) and on a group level using the Wilcoxon rank sum test (unpaired test). These non‐parametric tests were used since the sample size was small and not all data was normally distributed. *P* values under 0.05 were considered statistically significant, and *P* values under 0.001 are additionally highlighted in the figures.

## Results

All datasets were successfully acquired and analysed. For all participants, both atrial washout and atrial flow components were calculated and compared between groups.

### Velocity field in left heart

During sinus rhythm, the left atrium and appendage contract actively during late diastole (A‐wave in mitral valve flow).

**Figure 3:**
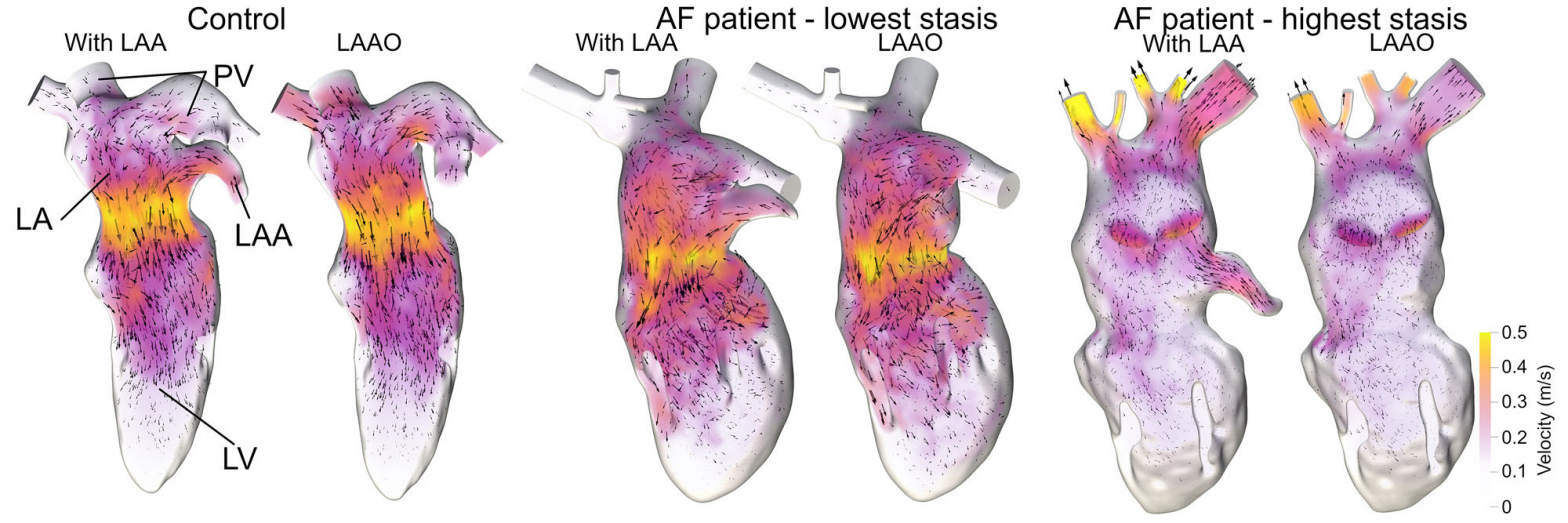
Velocity field of the left heart Velocity field of the left heart during late atrial contraction (A‐wave of mitral valve flow) for a control subject, the AF patient with the lowest residual volume and the AF patient with the highest residual volume.

Figure 3 shows the flow fields in the left heart during this timepoint for one control, the AF patient with the lowest stasis and the AF patient with the highest stasis during late atrial contraction. For the control and the AF patient with low stasis, before virtual occlusion, blood leaves the LAA in form of a jet directed towards the mitral valve. This jet is missing after LAA occlusion. In the control, LAAO leads to higher velocities in the pulmonary veins. Atrial velocities are much lower in the AF patient with high stasis and the jet from the LAA is directed towards the pulmonary veins. For this patient, the highest velocities are found in the pulmonary veins rather than at the mitral valve.

### Atrial blood flow washout

**Figure 4:**
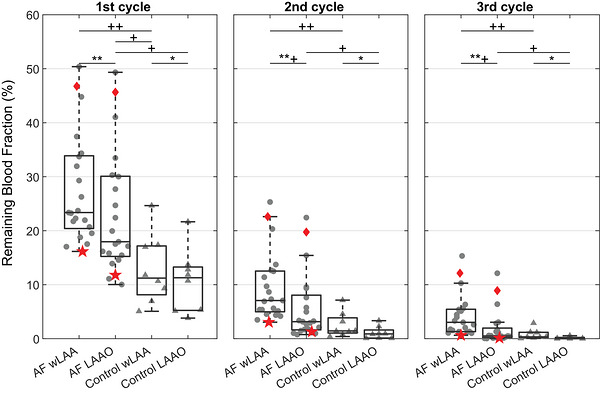
Remaining blood fraction in the left atrium Box plot of remaining blood fraction in the left atrium after 1, 2 and 3 cycles, respectively, in simulations with LAA (wLAA) and after occlusion (LAAO) for AF patients (circles) and controls (triangles). AF patient with highest residual volume from the component analysis is marked with a red diamond, AF patient with lowest residual volume is marked with red star. ^*^ *P* < 0.05, Wilcoxon signed rank test; ^**^ *P* < 0.001, Wilcoxon signed rank test; ^+^ *P* < 0.05, Mann–Whitney *U* test; ^++^ *P* < 0.001, Mann–Whitney *U* test. LAA, left atrial appendage.

The remaining blood fraction in the left atrium after 1, 2 and 3 cardiac cycles are presented in Fig. 4.

In the control group, on average, 11% of blood remained in the LA for more than one cardiac cycle. In the AF group with LAA, 23% of blood remained in the LA, while it was 18% after LAAO. LAAO significantly reduced the amount of blood staying in the LA for more than one cardiac cycle on an individual level (paired *t* test) in the AF group. The fraction of blood staying longer than three cardiac cycles was also significantly lower in a group wide comparison (Mann–Whitney *U* test). However, the fraction of blood staying for more than one and more than two cardiac cycles in the LA after LAAO was significantly higher in the AF group compared to controls. The AF patients shown in Figs 3 and 6 are highlighted in this figure using a red diamond and star for the patients with highest and lowest residual volume, respectively.

### Atrial flow component analysis

**Figure 5:**
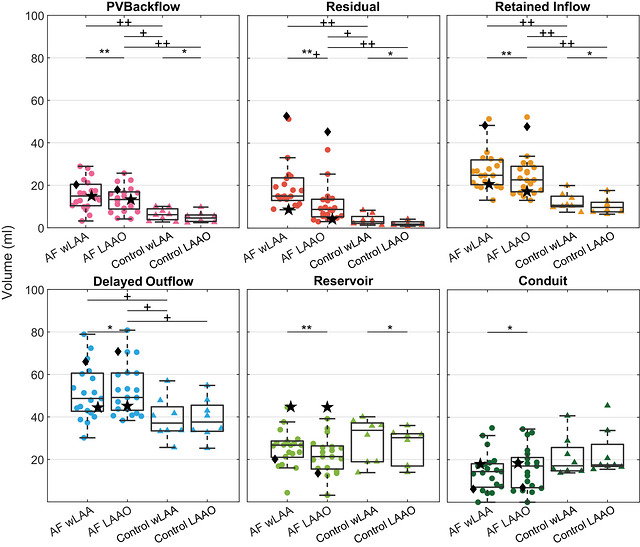
Volume of flow components in simulation with LAA (wLAA) and after occlusion (LAAO) Volume of flow components in simulation with LAA (wLAA) and after occlusion (LAAO) for AF patients (circles) and controls (triangles). AF patient with highest residual volume is marked with a black diamond, AF patient with lowest residual volume is marked with black star. ^*^ *P* < 0.05, Wilcoxon signed rank test; ^**^ *P* < 0.001, Wilcoxon signed rank test; ^+^ *P* < 0.05, Mann–Whitney *U* test; ^++^ *P* < 0.001, Mann–Whitney *U* test. LAA, left atrial appendage; PV, pulmonary veins.

Figure 5 shows the volume of each component when simulating the LA with LAA and after LAA occlusion for both the AF group and controls. *P*‐values of the comparison can be found in Table 2.

In both groups, the PV backflow, residual volume, retained inflow, and reservoir flow were significantly lower after LAAO, when using Wilcoxon signed rank test analysis. For the AF patients, the delayed outflow and conduit flow were significantly larger after LAAO. PV backflow, residual volume, retained inflow and delayed outflow remained significantly larger in the AF group compared to the controls after LAAO. The residual volume in the AF group was significantly lower after LAAO according to the Mann–Whitney *U* test as well.

While there is a significantly larger residual volume in the AF patients after LAAO compared to controls with LAA (*P* = 0.002), in many patients LAAO reduced the residual volume to levels similar to controls with LAA. In contrast, some AF patients have residual volumes of more than 30 ml after LAAO, more than four times higher than the largest residual volume of the controls with LAA.

### Spatial distribution of flow components

**Figure 6:**
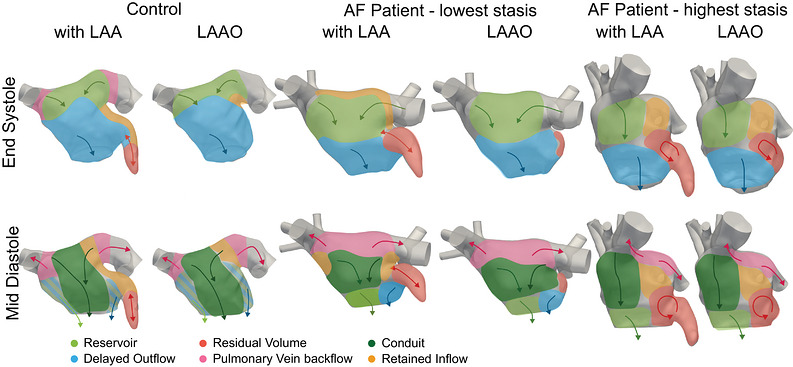
Schematic of the general position of the components at end systole and mid diastole Presented are one participant from the control group, the AF patient with lowest residual volume, and the AF patient with highest residual volume. LAA, left atrial appendage; LAAO, LAA occlusion

Figure 6 shows schematically where the different flow components are predominantly located.

At end systole, all reservoir flow has entered the left atrium, but the mitral valve has not opened yet. The reservoir component is primarily located close to the pulmonary veins at this time point, while delayed outflow is closer to the mitral valve. For the two AF patients, the dominant component in the LAA is the residual volume, while the LAA of the control also contains both retained inflow and residual volume. After LAAO, in the control there is no region of primarily residual volume in the LA. For the AF patient with the most residual volume, residual volume persists along the lateral side of the atrium even after LAAO. At mid‐diastole, the conduit flow travels through the left atrium. For the control and the AF patient with low stasis, conduit flow travels through the atrium along both the septal and lateral sides. For the AF patient with high stasis, conduit flow passes only along the septal side.

## Discussion

In this study, we investigated the effects of LAA occlusion on LA haemodynamics in paroxysmal AF patients in sinus rhythm using CFD simulations. We found that LAAO reduces stasis, in many cases to levels of the control group with the LAA. This was reflected in both decreased remaining blood fraction (on washout analysis) and residual volume (from component analysis), However, there are large individual variations, with some individuals still having residual volumes after LAAO that were four times larger than in the control group.

After virtual LAAO, we found a reduction in stasis, quantified by remaining blood fraction. This is in agreement with other CFD studies. D'Alessandro et al. compared two different types of LAA closure devices *vs*. no LAA removal, in five patients with ongoing atrial fibrillation. Atrial motion, in these patients with active fibrillation, was modelled by a random displacement field with small amplitudes. They found fewer remaining particles in the entire LA chamber after virtual LAAO (D'Alessandro et al., 2023). Zhong et al. (2024) investigated the haemodynamics in one patient who received LAAO for eight simulated LAAO scenarios and one pre‐LAAO model. The left atrial wall was assumed to be rigid. The authors found less remaining blood in the LA after the procedure compared to before. In the current study we investigate the effect of LAAO in two patient groups (AF *vs*. controls), all during sinus rhythm while considering the patient specific atrial wall motion. We show that LAAO can reduce stasis in the AF cohort to similar levels as in controls, but not for all individuals.

Stasis can be estimated using different parameters. In this study, remaining blood fraction (derived from washout analysis) and residual volume (derived from flow component analysis) were computed, while other studies use, for example, blood residence time (Bäck, Skoda et al., 2023) or residence time distribution (Sanatkhani et al., 2021). The advantage of the parameters used in this study is that they could be computed based on the CFD solution of the velocity field of one cardiac cycle. Thus, they require less computational resources than residence time calculations. However, they measure stasis after only a few cardiac cycles and it has not been fully investigated if this shorter analysis time is enough to reliably estimate thrombus formation. The main difference between remaining blood fraction and residual volume is that the remaining blood fraction is a relative measure, normalized by the atrial volume, while the residual volume is presented as the absolute volume of this component in millilitres. On average, the patients with AF had higher atrial volumes; therefore, the residual volume shows a significant difference between the AF patients after LAAO and the controls with LAA. In comparison, the remaining blood fraction after two cardiac cycles does not show a significant difference between these two groups. The patient with highest residual volume is shown in Figs 3 and 6 and marked with diamond in Figs 4 and 5. This patient does not have the highest remaining blood fraction; that distinction is held by a patient with a smaller atrial volume (Fig. 4). This patient has a smaller residual volume, but when normalized by the atrial volume, this patient has the highest remaining blood fraction. Thus, by presenting both the remaining blood fraction and residual volume, stasis can be analysed in different ways.

The remaining blood fraction after one cardiac cycle reduced on an individual level, but not on a group wide level and the patients with LAAO still had a significantly higher remaining blood fraction than the controls (both with LAA and after LAAO). After two and three cardiac cycles, the remaining blood fraction in AF patients reduced significantly after LAAO compared to before on a group wide level, with no significant difference to controls before LAAO. The increased stasis during the first cardiac cycle is probably caused by the generally enlarged atrial volume in the AF group, leading to slower velocities in the LA. The blood remaining longer in the LA is mainly located in the LAA and therefore reduced after LAAO.

Atrial flow component analysis provides information on the way LAAO changes the blood flow in the left atrium that cannot be estimated with stasis markers. Although LAAO reduces the overall atrial volume through removal of the LAA, the volume of delayed outflow and conduit volume increased on average. This could be related to the decreased reservoir flow. Reservoir flow enters the LA during ventricular systole. It does not reach the LAA, but the expanding LAA increases the inflow from the lungs during this time, allowing for more reservoir to enter. Thus, when the LAA is removed, less blood enters the LA during ventricular systole, lowering the reservoir flow. In the LAAO simulation, the ventricular stroke volume was not altered, thus the same amount of blood is passing through the mitral valve. This ventricular stroke volume is equal to the sum of conduit, reservoir and delayed outflow, which stays the same. When the reservoir flow decreases, conduit and delayed outflow need to increase to fulfil this condition. Both Asmarats et al. (2018) and Coisne et al. (2017) found that the ventricular stroke volume and the flow through the mitral valve stayed similar after LAAO. However, Coisne et al. (2017) found that after LAAO, the atrium got larger and contracted more. They hypothesized that this is due to an increased preload of the LA potentially caused by the reduced release of atrial natriuretic peptide after LAAO. In the current study, LAAO altered the blood flow in the entire atrium on a patient‐specific level, but the changes were not large enough to be significant in a group wide comparison. Thus, the changes are relatively small in relation to the differences between individuals. It is also not entirely clear how LAAO impacts the atrial and ventricular contraction patterns, and more research is needed on this.

LAA occlusion reduces but does not eliminate the risk of stroke. After LAA occlusion device implantation, device related thrombi sometimes occur (Van Gelder et al., 2024). Patients with surgical LAAO also remain at some risk for ischaemic stroke (Whitlock et al., 2021). Persistent amounts of stagnant blood in the LA may contribute to this ongoing risk. In this study we found that the amount of blood staying in the LA for more than three cycles was reduced after virtual LAAO. However, in some AF patients, this amount of blood was still more than two times larger than in the control group with intact LAA. These patients had a larger atrial volume and lower atrial ejection fraction compared to the other participants of the group. The residual volume after LAAO of the AF patient with high stasis in Fig. 6 is located on the lateral side, close to the LAA occlusion site. Since the patient was in sinus rhythm during the CT, the LAA was contracting; and LAAO reduced the velocities on the lateral atrial wall. Stagnating blood close to the LAA occlusion side could be a risk factor for developing device‐related thrombus. More research is needed to examine if this is also the case during active fibrillation and if these patients benefit less from LAAO.

There are some limitations in this study. The blood flow pattern in AF patients during sinus rhythm was compared to a control group without a diagnosis of AF. The control subjects were receiving a clinical CT examination and thus are not completely healthy. This group contained fewer subjects than the AF group and was not age or sex matched. Thus, some of the differences between the groups might be due to cofounders, for example, the participants of control group were younger than the AF group. In this study, the LAAO was idealized assuming optimal closure of the LAA. Actual LAAO placement might be different; for example, the occluder might sit further inside the LAA. This would lead to different blood flow patterns in reality compared to simulation. We found that the residual volume in the AF group was reduced after LAAO, in some patients to similar levels as the control group. However, the exact relation between the level of residual volume and other flow components and risk for thrombus formation or stroke is not fully understood. There is no established haemodynamic marker to predict stroke risk or thrombus formation, and this study used remaining blood fraction and flow component analysis. More studies are needed to establish reliable markers, as the optimal marker might differ between cardiovascular pathologies and conditions, as, for example, in AF patient in sinus rhythm, AF patients with ongoing AF arrhythmia, and patients with device‐related thrombi. Further, the degree and timing of atrial remodelling following LAAO is still unknown, and, in this study, we used a relatively simple approach, by keeping the atrial wall motion the same as before LAAO. Since the LAA is believed to be involved in atrial pressure regulation, LAAO might alter the atrial and ventricular contraction and filling patterns. In the future, CFD simulations could be used to investigate different remodelling patterns as well as investigating blood flow both during sinus rhythm and active fibrillation in the same patient and see their effect on atrial fluid dynamics.

In conclusion, for paroxysmal AF patients in sinus rhythm, LAAO can reduce blood stasis, indicated by a reduction of remaining blood fraction and residual volume, in some paroxysmal AF patients to levels comparable to the control group. However, the large variations among paroxysmal AF patients, with some still demonstrating four times higher stasis than controls after virtual LAAO, underscores the importance of accurate patient stratification.

## Additional information

## Competing interests

J.L., A.P., and T.E. are co‐founders of Cordicity. The other authors declare that they have no conflicts of interest.

## Author contributions

The patient data were acquired at Linköping University Hospital and analysed at Linköping University. S.B. was involved in study design, development of software, data investigation and analysis, visualization, and writing the original draft. J.L. was involved in study design, data analysis, supervision and providing research resources. I.S. and L.K. were involved in data acquisition. A.P. and C.C. were involved in data acquisition, supervision and providing research resources. T.E. was involved in study design, data analysis, supervision and providing research resources. All authors reviewed and edited the manuscript. All authors approved the final version of the manuscript; agree to be accountable for all aspects of the work in ensuring that questions related to the accuracy or integrity of any part of the work are appropriately investigated and resolved; and all persons designated as authors qualify for authorship, and all those who qualify for authorship are listed.

## Funding

This research was funded by Region Östergötland Grants 974839 and RÖ‐987498, Swedish Heart and Lung Foundation Grants 20200220 and 20230201, Swedish Research Council Grants 2018‐02779 and 2022‐03931, Sweden's Innovation Agency Vinnova Grant 2019‐02261, and Konung Gustaf V:s och Drottning Victorias Stiftelse. The computations were enabled by resources provided by the National Academic Infrastructure for Supercomputing in Sweden (NAISS), partially funded by the Swedish Research Council through grant agreement no. 2022‐06725 and by the National Supercomputer Centre (NSC), funded by Linköping University.

## Supporting information




Peer Review History

Supplementary Data

## Data Availability

All data supporting the results in the paper are listed in the Supplementary FigureData excel file.

## Data for Fig. 5

**Table 2:** *P* values for non‐parametric statistical comparisons in Fig. 5

	PV backflow	Residual	Retained	Delayed outflow	Reservoir	Conduit
AF wLAA *vs*. LAAO paired	**0.001**	**6.0 × 10^−5^**	**0.001**	**0.008**	**7 × 10^−5^**	**0.040**
AF wLAA *vs*. LAAO unpaired	0.339	**0.001**	0.279	0.744	0.113	0.554
Controls wLAA *vs*. LAAO paired	**0.023**	**0.008**	**0.008**	0.844	**0.016**	0.195
Controls wLAA *vs*. LAAO unpaired	0.279	0.130	0.442	0.959	0.328	0.505
AF LAAO *vs*. Control LAAO	**4.9 × 10^−4^**	**1.2 × 10^−4^**	**1.6 × 10^−4^**	**0.009**	0.317	0.213
AF LAAO *vs*. Controls wLAA	**0.004**	**0.002**	**4.0 × 10^−4^**	**0.008**	0.180	0.272
AF wLAA *vs*. Control wLAA	**0.001**	**4.6 × 10^−5^**	**1.9 × 10^−4^**	**0.016**	0.449	0.102

Values less than 0.05 are marked in bold.

### Simulation setup

The CFD simulations covered both the left atrium and left ventricle with patient‐specific wall motion using Ansys Fluent Version 19. Blood was assumed to be Newtonian with a viscosity of 3.5 × 10^−3^ Pa s and a density of 1060 kg/m^3^. The pulmonary veins were set as a constant relative pressure inlet, while the aorta was set as a pressure outlet. First, the systolic phase was simulated, where the mitral valve is closed and the aortic valve is open, followed by the diastolic phase where the aortic valve is closed and the mitral valve is open.

The motion of the endocardium was tracked as described in Bäck, Skoda et al. (2023). The code is available here: https://osf.io/bz89c/. This method deforms a triangulated surface based on 20 time‐resolved CT images covering the whole cardiac cycle. The position of each point in this surface was than smoothed in time and space using the spline fit function with 15 knots (Lundgren, 2022), and interpolated in time to a temporal resolution of 5 × 10^−4^ s. For each time step of the higher temporal resolution, the position of all points at this time was saved in a separate text file.

The code to set up the fluent files can be found here: https://gitlab.liu.se/cim_public/atrialcfd_fluentexample. In Fluent, the moving walls were modelled using the dynamic mesh approach with a user‐defined function based on the ‘define grid motion’ macro. At each time step, the position file of the previous time step and the current time step were loaded. Then for each surface node of the CFD mesh, the 5 closest points in the position file of the previous time step were found, and their motion was calculated as the difference between the current position and the previous position. The location of the surface node was then updated based on the inversed weighted distances of the 5 closest points. In the dynamic mesh settings, the smoothing setting was active, using the diffusion method.

Due to the large change in volume, the new meshes were generated every 50 time‐steps (0.025 s) during an initial mesh setup cycle. The meshes were set up so that elements were smaller closer to the wall and larger in the centre of the domain. The smallest elements had an edge length of 0.25 mm, and the largest elements had an edge length of 2 mm. Overall, the meshes contained between 5 and 12 million cells.

The simulations were conducted using second order accuracy and the Piso scheme. The convergence criteria was 1 × 10^−4^ Pa s.

The simulations including the LAA were run for five cardiac cycles, and the solution of the following cardiac cycle was exported at a resolution of 0.025 s.

For the LAAO simulations, the LAA was digitally removed, and no alterations were made to the motion of the LA or the LV. This way, the ventricular stroke volume remained the same, but the pulmonary vein flow was slightly different. The solution with the LAA after six cardiac cycles was used as an initial condition and the flow field was simulated for another two full cardiac cycles and one systolic phase. The last full cardiac cycle containing a diastolic and systolic phase was exported with a resolution of 0.025 s and analysed.

## Washout analysis

For the washout analysis, the LA was seeded with particles at end ventricular systole. The particles were placed on an isotropic Cartesian grid with a resolution of 1 mm, so each particle represented a volume of 1 mm^3^. The motion of the particles was traced using the particle path filter in ParaView version 5.11. This filter integrates the velocity field with the 4th order Runge‐Kutta method. Each particle was traced for three cardiac cycles, by looping the exported velocity field of one cardiac cycle three times and it was registered when the particle left the left atrium. Then the percentage of particles still in the LA after 1, 2, and 3 cardiac cycles was calculated.

## Flow component analysis

To capture the atrial blood flow, two different techniques for particle tracking were combined. The atrial volume at end ventricular systole (when it is the largest) was seeded with particles structured on an isotropic Cartesian grid with a resolution of 2 mm, so that each particle represents a volume of 0.008 ml. These particles were traced both backwards and forward in time for one cardiac cycle each using the particle path filter in ParaView version 5.11. From this method, five components were derived.

**Table:**

	Enters in systole	Enters in diastole	Already in LA
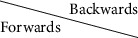
Leaves MV (during diastole)	**Reservoir**	**Delayed outflow**	
Stays	**Retained inflow**		**Residual volume**
Leaves PV	**Pulmonary vein backflow**		

During ventricular systole, the mitral valve is open and there is the possibility for blood to enter the LA through the pulmonary veins and leave through the mitral valve, or flow back towards the lungs. This blood is not captured by the previously described method, since it is not in the LA during end ventricular systole. To capture the volume of this blood, it is tracked through particle seeding at the pulmonary veins. These particles were released from a plane at the inlet of each pulmonary vein, with an isotropic grid spacing of 1 mm, and new particles were released every 0.025 s. Thus, each particle represented a volume calculated by eqn (1):

(1)
$$V_{\mathrm{particle}}=A\;\times \;v_{\mathrm{norm}}\;\times dt.$$

A is the area each particle represents (1 mm^2^), $v_{\mathrm{norm}}$ is the velocity of the particle normal to the seeding plane and dt is the time step of 0.025 s. Particles entering the LA during ventricular diastole and leaving through the mitral valve in the same phase are referred to as conduit flow. Particles entering the LA but leaving through the pulmonary veins were added to the pulmonary vein backflow.
