# Supplementary material for: Virtual left atrial appendage occlusion in paroxysmal atrial fibrillation during sinus rhythm predicts variable reductions in blood stasis
Source: J Physiol. 2025 Jul 10;604(13):5342–54. doi: 10.1113/JP288587 (PMC13327759; doi:10.1113/JP288587)
Supplement: Supplementary file 1 — Peer Review History [file TJP-604-5342-s002.pdf]

The Journal of Physiology

Virtual Left Atrial Appendage Occlusion in Paroxysmal Atrial Fibrillation during Sinus Rhythm predicts Variable Reductions in Blood Stasis

Sophia Bäck, Jonas Lantz, Iulia Skoda, Lars O Karlsson, Anders Persson, Carl-Johan Carlhäll, and Tino Ebbers
DOI: 10.1113/JP288587

Corresponding author(s): Tino Ebbers (tino.ebbers@liu.se)

The referees have opted to remain anonymous.

Review Timeline:

Submission Date:	30-Jan-2025
Editorial Decision:	14-Feb-2025
Revision Received:	16-Apr-2025
Editorial Decision:	28-Apr-2025
Revision Received:	28-May-2025
Editorial Decision:	30-May-2025
Revision Received:	17-Jun-2025
Accepted:	18-Jun-2025

Senior Editor: Bjorn Knollmann

Reviewing Editor: Axel Loewe

Transaction Report:

(Note: With the exception of the correction of typographical or spelling errors that could be a source of ambiguity, letters and reports are not edited. Depending on transfer agreements, referee reports obtained elsewhere may or may not be included in this compilation. Referee reports are anonymous unless the Referee chooses to sign their reports.)

Dear Dr Ebbers,

Re: JP-RP-2025-288587 "Virtual Left Atrial Appendage Occlusion Reduces Stasis in Paroxysmal Atrial Fibrillation Patients during Sinus Rhythm" by Sophia BÃ¤ck, Jonas Lantz, Iulia Skoda, Lars O Karlsson, Anders Persson, Carl-Johan Carlhäll, and Tino Ebbers

Thank you for submitting your manuscript to The Journal of Physiology. It has been assessed by a Reviewing Editor and by 2 expert referees and we are pleased to tell you that it is potentially acceptable for publication following satisfactory major revision.

Please advise your co-authors of this decision as soon as possible.

The referee reports are copied at the end of this email.

Please address all the points raised and incorporate all requested revisions or explain in your Response to Referees why a change has not been made. We hope you will find the comments helpful and that you will be able to return your revised manuscript within 2 months. If you require longer than this, please contact journal staff: jp@physoc.org. Please note that this letter does not constitute a guarantee for acceptance of your revised manuscript.

Your revised manuscript should be submitted online using the link in your Author Tasks: Link Not Available. This link is accessible via your account as Corresponding Author; it is not available to your co-authors. If this presents a problem, please contact journal staff (jp@physoc.org). Image files from the previous version are retained on the system. Please ensure you replace or remove any files that are being revised.

If you do not wish to submit a revised version of your manuscript, you must inform our journal staff (jp@physoc.org) or reply to this email to request withdrawal. Please note that a manuscript must be formally withdrawn from the peer review process at one journal before it may be submitted to another journal.

TRANSPARENT PEER REVIEW POLICY: To improve the transparency of its peer review process, The Journal of Physiology publishes online as supporting information the peer review history of all articles accepted for publication. Readers will have access to decision letters, including Editors' comments and referee reports, for each version of the manuscript, as well as any author responses to peer review comments. Referees can decide whether or not they wish to be named on the peer review history document.

ABSTRACT FIGURES: Authors are expected to use The Journal's premium BioRender account to create/redraw their Abstract Figures. Information on how to access this account is here: https://physoc.onlinelibrary.wiley.com/journal/14697793/biorender-access.

This will enable Authors to create and download high-resolution figures. If authors have used the free BioRender service, they can use the instructions provided in the link above to download a high-resolution version suitable for publication.

The link provided should only be used for the purposes of this submission. Authors will be charged for figures created on this account if they are not related to this manuscript submission.

LANGUAGE EDITING AND SUPPORT FOR PUBLICATION: If you would like help with English language editing, or other article preparation support, Wiley Editing Services offers expert help, including English Language Editing, as well as translation, manuscript formatting, and figure formatting at www.wileyauthors.com/eeo/preparation. You can also find resources for Preparing Your Article for general guidance about writing and preparing your manuscript at www.wileyauthors.com/eeo/prepresources.

RIGOUR AND REPRODUCIBILITY: We recommend authors consult The Journal's Rigour and Reproducibility requirements to ensure that their manuscript is suitable for publication: https://physoc.onlinelibrary.wiley.com/pb-assets/hub-assets/physoc/documents/TJP-Rigour-and-Reproducibility-Requirements.pdf

REVISION CHECKLIST:

Check that your Methods section conforms to journal policy: https://jp.msubmit.net/cgi-bin/main.plex?form_type=display_requirements#methods.

Check that data presented conforms to the statistics policy: https://jp.msubmit.net/cgi-bin/main.plex?form_type=display_requirements#statistics.

Upload a full Response to Referees file. To create your 'Response to Referees': copy all the reports, including any comments from the Senior and Reviewing Editors, into a Microsoft Word, or similar, file and respond to each point, using font or background colour to distinguish comments and responses and upload as the required file type.

Please upload two versions of your manuscript text: one with all relevant changes highlighted and one clean version with no changes tracked. The manuscript file should include all tables and figure legends, but each figure/graph should be uploaded as separate, high-resolution files.

You may also upload:

- 'Potential Cover Art' for consideration as the issue's cover image
- Appropriate Supporting Information (Video, audio or data set: see https://jp.msubmit.net/cgi-bin/main.plex?form_type=display_requirements#supp).

We look forward to receiving your revised submission.

If you have any queries, please reply to this email and we will be pleased to advise.

Yours sincerely,

Bjorn Knollmann
Senior Editor
The Journal of Physiology

REQUIRED ITEMS

- Author photo and profile. First or joint first authors are asked to provide a short biography (no more than 100 words for one author or 150 words in total for joint first authors) and a portrait photograph. These should be uploaded and clearly labelled together in a Word document with the revised version of the manuscript. See Information for Authors for further details.

- You must start the Methods section with a paragraph headed Ethical Approval. If experiments were conducted on humans, confirmation that informed consent was obtained, preferably in writing, that the studies conformed to the standards set by the latest revision of the Declaration of Helsinki and that the procedures were approved by a properly constituted ethics committee, which should be named, must be included in the article file. If the research study was registered (clause 35 of the Declaration of Helsinki), the registration database should be indicated, otherwise the lack of registration should be noted as an exception (e.g. The study conformed to the standards set by the Declaration of Helsinki, except for registration in a database). For further information see: https://physoc.onlinelibrary.wiley.com/hub/human-experiments.

- Your manuscript must include a complete Additional Information section, including competing interests; funding; author contributions and acknowledgements.

- Please upload separate high-quality figure files via the submission form.

- A Data Availability Statement is required for all papers reporting original data. This must be in the Additional Information section of the manuscript itself. It must have the paragraph heading 'Data Availability Statement'. All data supporting the results in the paper must be either: in the paper itself; uploaded as Supporting Information for Online Publication; or archived in an appropriate public repository. The statement needs to describe the availability or the absence of shared data. Authors must include in their statement: a link to the repository they have used, or a statement that it is available as Supporting Information; reference the data in the appropriate sections(s) of their manuscript; and cite the data they have shared in the References section. Whenever possible, the scripts and other artefacts used to generate the analyses presented in the paper should also be publicly archived. If sharing data compromises ethical standards or legal requirements then authors are not expected to share it, but must note this in their statement. For more information, see our Statistics Policy.

- Please include an Abstract Figure file, as well as the Figure Legend text within the main article file. The Abstract Figure is a piece of artwork designed to give readers an immediate understanding of the research and should summarise the main conclusions. If possible, the image should be easily 'readable' from left to right or top to bottom. It should show the physiological relevance of the manuscript so readers can assess the importance and content of its findings. Abstract Figures should not merely recapitulate other figures in the manuscript. Please try to keep the diagram as simple as possible and without superfluous information that may distract from the main conclusion(s). Abstract Figures must be provided by authors

no later than the revised manuscript stage and should be uploaded as a separate file during online submission labelled as File Type 'Abstract Figure'. Please also ensure that you include the figure legend in the main article file. All Abstract Figures should be created using BioRender. Authors should use The Journal's premium BioRender account to export high-resolution images. Details on how to use and access the premium account are included as part of this email.

- Please include a full title page as part of your main article (Word) file, which should contain the following: title, authors, affiliations, corresponding author name and contact details, keywords, and running title.

EDITOR COMMENTS

Reviewing Editor: Methods Details:
Computational setup missing (see specific comments below).

Ethics Concerns:
Statement about conforming with the Declaration of Helsinki is missing.

Comments to the Author:

Your manuscript was reviewed by two expert referees who acknowledge that this is an interesting study addressing a relevant question. In their reports, both referees point out a number of points that need to be addressed for further consideration of this manuscript.

In addition to the points raised by the reviewers, please provide more details on technical aspects necessary for reproducibility. For example: the time resolution of your imaging data is in the order of 50ms (5% of RR) whereas the simulation time step was 0.5ms. How were the boundary conditions interpolated between image frames?

A data availability statement is required as per the author guidelines: https://jp.msubmit.net/cgi-bin/main.plex?form_type=display_requirements#addinfo

Simulation parameters need to be made available as per the author guidelines: https://jp.msubmit.net/cgi-bin/main.plex?form_type=display_requirements#modelling

Please also see 'Required Items' above.

Senior Editor:

I concur with the reviewing editor's assessment.

REFEREE COMMENTS

Referee #1:

Dear Authors,

Thank you for the opportunity to review your manuscript. I appreciate the effort and dedication you have put into this important work. I have carefully read through your study and have a few suggestions and clarifications that I believe will strengthen your manuscript and improve its clarity and impact. Please find my detailed comments attached.

[FILE ATTACHED]

Referee #2:

Back et al, in this study, aimed to evaluate if paroxysmal AF patients, who are early in the disease and still intermittently in sinus rhythm, could benefit from LAAO using flow assessment by estimating atrial flow stasis. The authors noted that these patients are not considered for LAAO in clinical practice and the deployment of the LAAO device is virtual. The study has several strengths including patient-specific simulations with implementation of wall motion, and the novelty of washout analysis in the context of the left atrium in relation to LAAO. However, there are several concerns that I detail in the comments below:

- How come the cohort that the authors compared was not balanced? and how does this imbalance affect the results of this study? In other words, if controls' numbers were to be increased to match the AF numbers, would the authors still obtain the same results?

- Why uncontrolled hypertension was considered an exclusion criterion?

- How were the paroxysmal AF patients selected? As the authors pointed out, paroxysmal AF patients can still be in sinus rhythm upon clinical examination. Were the patients selected in AF at the moment of imaging?

- Was it a laminar flow regime that was selected?

- This reviewer did not quite understand why the LAAO simulations were run for 2.5 cardiac cycles? That's a very weird number to set the simulations.

- What were the inlet boundary conditions used?

- Although the authors reference their previous work on their simulation methodology, for completion of this paper, a few sentences describing the methods are necessary.

- It is not clear how the particle seeding at the beginning was initialized.

- For particle tracking analysis, how come only 2 cycles were considered? and for the LAAO cases, was the same duration considered?

- The currently described methods for particle tracking are not clear, specifically how the particles that went into the PVs for example were tracked, and what about leftover particles from a previous cardiac cycle? Overall, some more explanation is needed.

- How was the residual volume calculated? And all the parameters referenced in Figure 5. I recommend that the authors add these mathematical formulas and explanations to the manuscript for completion and if not into the body of the main text, in supplementary or appendix.

- The differentiation numerically speaking between all the defined "flows" such as reservoir flow, etc. needs to be more elaborated on.

- Regarding statistics, were the distributions Gaussian? if not, a non-parametric test is needed for accurate comparisons.

- In Figure 3, what phase is that?

- The authors mention "During sinus rhythm, the left atrium and appendage contract actively during late diastole (A wave in mitral valve flow)", so for these AF patients the atrial kick was still there? and if this is the case, what would differentiate these patients from control?

- In the results of Figure 4, it is not clear how the third cycle results were computed for the LAAO cases, if they were only simulated for 2.5 cardiac cycles.

- The discussion should focus on the significance of the findings. For example, how can we interpret a 5% reduction in flow stasis? Is it meaningful when it comes to the evaluation of thrombosis potential?

- The discussion does not include any comment about the statistical insignificance derived from unpaired comparisons suggesting high inter-patient variability.

- The authors state that LAAO reduces stasis, but the results show that some patients have higher stasis after LAAO compared with controls.

- The results show that for some patients, LAAO does not yield favorable results in terms of improvement of hemodynamics. So these patients should not receive LAAO?

- The authors mention that LAAO could be considered for paroxysmal AF patients but they do neither evaluate nor discuss any clinical endpoints such as stroke reduction or thrombus formation. This is a huge drawback and needs to be addressed for the completion of the study. This makes this statement overreaching.

- The authors do not assess thrombus risk directly and therefore their statement that LAAO reduces stroke risk is far fetched.

- The limitations section should include that the occlusion was idealized while in reality devices may not have been placed in a manner that would be similar to how the authors assumed it to be.

END OF COMMENTS

Dear Authors,

Thank you for the opportunity to review your manuscript. I appreciate the effort and dedication you have put into this important work. I have carefully read through your study and have a few suggestions and clarifications that I believe will strengthen your manuscript and improve its clarity and impact. Please find my detailed comments below.

Line 42-53: A reader might learn from this paragraph that AF management only involves anticoagulation and LAA occlusion, while this is not true. Please revise accordingly.

Line 54-57: Some statistics are given here but needs clarifications.

1) *"LAAO reduces the risk for stroke"* How much does it reduce the stroke compared to patients who don't
2) *"A large randomized controlled trial found that surgical LAA removal reduces ischemic strokes from 6.9% to 4.6% (Whitlock et al., 2021)"* Reader would think that in that study all the LAA were surgically removed, however, in that study only 55.7% of participant had their LAA cut and sewed while for the rest some sort of occlusion was done.

Line 121-123: Please describe the boundary conditions at the pulmonary veins and mitral valve as well.

Line 127-128: In this study Newtonian flow assumption has been used. This needs some justifications with references. Many studies have argued that non-Newtonian flow simulations are necessary. Recently, https://doi.org/10.3389/fcvm.2023.1070498 showed that while non-Newtonian assumption would lead to slower blood washout, the choice of blood rheology model does not affect the rank ordering among the study subjects. Therefore, use of Newtonian model is justified in this case.

Line 128-132: What was the test and criteria that has been used to determine 6 cardiac cycles are sufficient to reach steady state? Study https://doi.org/10.3389/fphys.2021.633135 and https://doi.org/10.3389/fcvm.2023.1070498 has used 25 cycles. While https://doi.org/10.1007/s10439-016-1590-x has only used 2 cycles.

Also how many cycles were used as an initial condition for the case with the LAA?

Line 131: 2.5 cardiac cycles were simulated. What was the criteria again? This study https://doi.org/10.3390/math9182304 sensitivity analysis for their simulations and have suggested up to 15 cycles, Similarly, this study https://doi.org/10.3389/fcvm.2023.1070498 has done a analysis and suggested 20,000

seconds. Please show your justifications here or in other sections with a discussion why 2.5 cycles are enough without directing readers to look into your previous publications.

Line 270-271: As it is pointed out by the authors (Line 318-322) themselves, analyzing the patient-specifics is important. Readers would be interested to know about the characteristics and details of the individuals whose stasis level did not reduce after LAAO. Please discuss.

Discussion: Please in a paragraph discuss how the results from the flow component analysis varies from the washout analysis. Further, in the Methods section it is pointed out that "*residual volume, which here was considered as a marker for stasis*". Explain how the information given by residual volume is different from the blood washout and further how is it compare to other metrics such as residence time distribution in the literature. In what ways each of these methods are superior and your recommendation for researchers.

Do other components (retained inflow, delayed outflow, etc.) add any extra information about stasis that is not already given by residual volume, blood washout or residence time distribution?

Comments from Editor

REQUIRED ITEMS

1. Author photo and profile. First or joint first authors are asked to provide a short biography (no more than 100 words for one author or 150 words in total for joint first authors) and a portrait photograph. These should be uploaded and clearly labelled together in a Word document with the revised version of the manuscript. See Information for Authors for further details.
   We uploaded this document.
2. You must start the Methods section with a paragraph headed Ethical Approval. If experiments were conducted on humans, confirmation that informed consent was obtained, preferably in writing, that the studies conformed to the standards set by the latest revision of the Declaration of Helsinki and that the procedures were approved by a properly constituted ethics committee, which should be named, must be included in the article file. If the research study was registered (clause 35 of the Declaration of Helsinki), the registration database should be indicated, otherwise the lack of registration should be noted as an exception (e.g. The study conformed to the standards set by the Declaration of Helsinki, except for registration in a database). For further information see: https://physoc.onlinelibrary.wiley.com/hub/human-experiments.

   An Ethical Approval section was added to the manuscript in accordance to the guidelines.

3. - Your manuscript must include a complete Additional Information section, including competing interests; funding; author contributions and acknowledgements.
   We added this section to the manuscript.

4. - Please upload separate high-quality figure files via the submission form.
   We uploaded the figures separately.

5. - A Data Availability Statement is required for all papers reporting original data. This must be in the Additional Information section of the manuscript itself. It must have the paragraph heading 'Data Availability Statement'. All data supporting the results in the paper must be either: in the paper itself; uploaded as Supporting Information for Online Publication; or archived in an appropriate public repository. The statement needs to describe the availability or the absence of shared data. Authors must include in their statement: a link to the repository they have used, or a statement that it is available as Supporting Information; reference the data in the appropriate sections(s) of their manuscript; and cite the data they have shared in the References section. Whenever possible, the scripts and other artefacts used to generate the analyses presented in the paper should also be publicly archived. If sharing data compromises ethical standards or legal requirements then authors are not

expected to share it, but must note this in their statement. For more information, see our Statistics Policy.
See point 3

6. - Please include an Abstract Figure file, as well as the Figure Legend text within the main article file. The Abstract Figure is a piece of artwork designed to give readers an immediate understanding of the research and should summarise the main conclusions. If possible, the image should be easily 'readable' from left to right or top to bottom. It should show the physiological relevance of the manuscript so readers can assess the importance and content of its findings. Abstract Figures should not merely recapitulate other figures in the manuscript. Please try to keep the diagram as simple as possible and without superfluous information that may distract from the main conclusion(s). Abstract Figures must be provided by authors no later than the revised manuscript stage and should be uploaded as a separate file during online submission labelled as File Type 'Abstract Figure'. Please also ensure that you include the figure legend in the main article file. All Abstract Figures should be created using BioRender. Authors should use The Journal's premium BioRender account to export high-resolution images. Details on how to use and access the premium account are included as part of this email.
We uploaded an Abstract Figure. It was not possible to create the figure using BioRenderer, since the program did not include and figures on the left atrial appendage or left atrial appendage occlusion.
7. - Please include a full title page as part of your main article (Word) file, which should contain the following: title, authors, affiliations, corresponding author name and contact details, keywords, and running title.
We have updated the title page.
8. Reviewing Editor: Methods Details:
Computational setup missing (see specific comments below).
We added a file with supplementary information on the simulation setup.

Ethics Concerns:

We added a section on Ethical Approval to the Methods.

9. Statement about conforming with the Declaration of Helsinki is missing.
We added this to the Ethical Approval section.

10. In addition to the points raised by the reviewers, please provide more details on technical aspects necessary for reproducibility. For example: the time resolution of your imaging data is in the order of 50ms (5% of RR) whereas the simulation time step was 0.5ms. How were the boundary conditions interpolated between image frames?
The boundary conditions were interpolated in time by fitting a spline through the locations of each surface point during the cardiac cycle. We have clarified this in the supplementary info document.

11. A data availability statement is required as per the author guidelines:
    https://jp.msubmit.net/cgi-bin/main.plex?form_type=display_requirements#addinfo
    We added a Data Availability statement.

12. Simulation parameters need to be made available as per the author guidelines:
    https://jp.msubmit.net/cgi-bin/main.plex?form_type=display_requirements#modelling
    'As in any experimental study, results from the model should add new scientific insights. The model should be based on physiological data where the parameters can be directly linked to experimental data. The model should be described in such a way that the results can be reproduced, i.e. all parameters are listed and any computation codes made available. Where possible there should be a detailed sensitivity analysis of the conclusions reached against the parameters proposed within the bounds of the data used to constrain them. Papers that develop integrative physiological models which link functions together or observations across scales would be encouraged.'
    We added a supplementary info file to the manuscript, explaining the computational setup in more detail.

Reviewer 1

Dear Authors, Thank you for the opportunity to review your manuscript. I appreciate the effort and dedication you have put into this important work. I have carefully read through your study and have a few suggestions and clarifications that I believe will strengthen your manuscript and improve its clarity and impact. Please find my detailed comments below.

Line 42-53: A reader might learn from this paragraph that AF management only involves anticoagulation and LAA occlusion, while this is not true. Please revise accordingly.

We have clarified this in the first paragraph.

Line 54-57: Some statistics are given here but needs clarifications. 1) "LAAO reduces the risk for stroke" How much does it reduce the stroke compared to patients who don't 2) "A large randomized controlled trial found that surgical LAA removal reduces ischemic strokes from 6.9% to 4.6% (Whitlock et al., 2021)" Reader would think that in that study all the LAA were surgically removed, however, in that study only 55.7% of participant had their LAA cut and sewed while for the rest some sort of occlusion was done.

We clarified in the first sentence of this paragraph that LAAO reduces stroke risk to similar levels as oral anticoagulants. We have rephrased the paragraph, so it says LAA occlusion or surgical LAA removal.

Line 121-123: Please describe the boundary conditions at the pulmonary veins and mitral valve as well.

We clarified this in the methods:

This motion was set as the boundary condition at the walls, while zero relative pressure was prescribed at the pulmonary veins and the ascending aorta. The mitral valve and aortic valve were set to either fully open or closed. The flow rate through the mitral valve was an implicit result of including the ventricular motion in the simulation.

Line 127-128: In this study Newtonian flow assumption has been used. This needs some justifications with references. Many studies have argued that non-Newtonian flow simulations are necessary. Recently, https://doi.org/10.3389/fcvm.2023.1070498 showed that while non-Newtonian assumption would lead to slower blood washout, the choice of blood rheology model does not affect the rank ordering among the study subjects. Therefore, use of Newtonian model is justified in this case.

We clarified this in the methods.

Line 128-132: What was the test and criteria that has been used to determine 6 cardiac cycles are sufficient to reach steady state? Study https://doi.org/10.3389/fphys.2021.633135 and https://doi.org/10.3389/fcvm.2023.1070498 has used 25 cycles. While https://doi.org/10.1007/s10439-016-1590-x has only used 2 cycles. Also how many cycles were used as an initial condition for the case with the LAA?

We clarified the computational setup. The CFD simulations with the LAA were simulated for 6 cardiac cycles. The velocity field of the last cardiac cycle, which represents a converged velocity field was used to calculate the washout and the flow components, by looping this cardiac cycle .

Line 131: 2.5 cardiac cycles were simulated. What was the criteria again? This study https://doi.org/10.3390/math9182304 sensitivity analysis for their simulations and have suggested up to 15 cycles, Similarly, this study https://doi.org/10.3389/fcvm.2023.1070498 has done a analysis and suggested 20,000 seconds. Please show your justifications here or in other sections with a discussion why 2.5 cycles are enough without directing readers to look into your previous publications.

The CFD simulations in the LAAO were initialized with the solution with the LAA and then run for 2 full cardiac cycles, and an additional systolic phase. The solution of the last diastolic + systolic phase were then looped for 3 cardiac cycles in the washout analysis and 2 cardiac cycles in the flow component analysis. In contrast to the mentioned publications, we do not aim to calculate the converged, time independent remaining volume, to reduce the computational cost. By comparing the results from the AF group to controls, we could show that already this simpler method is able to identify differences. We added a section to the discussion on the comparison of our markers to residence time parameters.

Line 270-271: As it is pointed out by the authors (Line 318-322) themselves, analyzing the patient-specifics is important. Readers would be interested to know about the characteristics and details of the individuals whose stasis level did not reduce after LAAO. Please discuss.

The main characteristic distinguishing between these individuals is a larger atrial volume, especially in relation to the overall cardiac size. We added this info to the discussion.

Discussion: Please in a paragraph discuss how the results from the flow component analysis varies from the washout analysis. Further, in the Methods section it is pointed out that "residual volume, which here was considered as a marker for stasis". Explain how the information given by residual volume is different from the blood washout and further how is it compare to other metrics such as residence time distribution in the literature. In what ways each of these methods are superior and your recommendation for researchers.

We have added this paragraph to the discussion (Paragraph 3).

Do other components (retained inflow, delayed outflow, etc.) add any extra information about stasis that is not already given by residual volume, blood washout or residence time distribution?

Yes, these components allow for a more comprehensive understanding of the atrial blood flow beyond stasis. We have clarified this in the 5th paragraph of the discussion.

Reviewer 2

Back et al, in this study, aimed to evaluate if paroxysmal AF patients, who are early in the disease and still intermittently in sinus rhythm, could benefit from LAAO using flow assessment by estimating atrial flow stasis. The authors noted that these patients are not considered for LAAO in clinical practice and the deployment of the LAAO device is virtual. The study has several strengths including patient-specific simulations with implementation of wall motion, and the novelty of washout analysis in the context of the left atrium in relation to LAAO. However, there are several concerns that I detail in the comments below:

1. - How come the cohort that the authors compared was not balanced? and how does this imbalance affect the results of this study? In other words, if controls' numbers were to be increased to match the AF numbers, would the authors still obtain the same results?

   Unfortunately, the control participants were collected separately from the patients with AF, and therefore the groups were not balanced. We have added this to the limitations of this study:.

   > This group contained fewer subjects than the AF group and was not age or sex matched. Thus, some of the differences between the groups might be due to cofounders, for example, the participants of control group were younger than the AF group.

2. - Why uncontrolled hypertension was considered an exclusion criterion?

   Uncontrolled hypertension was an exclusion criterion, since high pressure in the aorta would affect the hemodynamics in the entire left heart. We clarified this in the manuscript in the patient characteristics section.
3. - How were the paroxysmal AF patients selected? As the authors pointed out, paroxysmal AF patients can still be in sinus rhythm upon clinical examination. Were the patients selected in AF at the moment of imaging?

For this study, paroxysmal AF patients were selected that received a clinical CT exam before catheter ablation. The AF patients were in sinus rhythm during the examination. We have clarified this in the Patient characteristics and image acquisition section and in the aim.

4. - Was it a laminar flow regime that was selected?

   Yes, we added this information to the methods section.

5. - This reviewer did not quite understand why the LAAO simulations were run for 2.5 cardiac cycles? That's a very weird number to set the simulations.

   We clarified this in the methods section of the manuscript:

   For the virtual LAAO occlusion, the solution with the LAA after 6 cardiac cycles was used as an initial condition and the flow field was simulated for another two full cardiac cycles and one systolic phase. The last full cardiac cycle containing a diastolic and systolic phase was analyzed, which had a converged velocity field.

6. - What were the inlet boundary conditions used?

   - Although the authors reference their previous work on their simulation methodology, for completion of this paper, a few sentences describing the methods are necessary.
   - It is not clear how the particle seeding at the beginning was initialized.
   - For particle tracking analysis, how come only 2 cycles were considered? and for the LAAO cases, was the same duration considered?

   We have revised our method section and added an additional document with supplementary information, where we describe the method in more detail. The pulmonary veins were defined as constant relative pressure inlets. Since the CFD simulations included both the LA and the LV with patient-specific endocardial motion, this motion drove the flow and defined the flow rate at the pulmonary veins.

   The particle seeding was done based on the converged velocity field of one cardiac cycle, which was then looped two times for the flow component analysis and three times for the washout study. This was done in the same manner for both the case with LAA and after LAAO.

   However, the CFD simulations were run differently long for the case with LAA and after LAAO. For the simulation with LAA, the sixth cardiac cycle was analysed. This cardiac cycle was also used as an initial condition for the LAAO case, which was run for two full cardiac cycles plus one systolic phase. The last full cardiac cycle, containing first a diastolic and then a systolic phase was analysed.

7. The currently described methods for particle tracking are not clear, specifically how the particles that went into the PVs for example were tracked, and what about leftover particles from a previous cardiac cycle? Overall, some more explanation is needed.

   - How was the residual volume calculated? And all the parameters referenced in Figure 5. I recommend that the authors add these mathematical formulas and explanations to the manuscript for completion and if not into the body of the main text, in supplementary or appendix.
   - The differentiation numerically speaking between all the defined "flows" such as reservoir flow, etc. needs to be more elaborated on.

In the supplementary information that we added to the manuscript, we describe the particle tracking in more detail. There, we describe how the particles were seeded and how we defined the flows and their volumes, based on the path taken by the particles.

8. - Regarding statistics, were the distributions Gaussian? if not, a non-parametric test is needed for accurate comparisons.
   Thank you for this comment, we noticed that some parameters were not gaussian distributed. We changed the statistical analysis to nonparametric tests. This altered the p values slightly, but not substantially, so that all conclusions remained the same.

9. In Figure 3, what phase is that?
   This figure is during late atrial contraction (A-wave of mitral valve flow). We added this info to the figure caption.

10. The authors mention "During sinus rhythm, the left atrium and appendage contract actively during late diastole (A wave in mitral valve flow)", so for these AF patients the atrial kick was still there? and if this is the case, what would differentiate these patients from control?
    Yes, these patients are at an early stage of AF, and were in sinus rhythm during the CT exam. As described in the results text, the patient with highest stasis had very low atrial contraction, but for the AF patient with lowest stasis, the pattern looks more like the control. While still being in sinus rhythm, what differentiates the AF patients with high stasis is that they have increased atrial volumes and reduced atrial ejection fraction. We clarified the description of the patients with high stasis in the 6th paragraph of the discussion.
11. - In the results of Figure 4, it is not clear how the third cycle results were computed for the LAAO cases, if they were only simulated for 2.5 cardiac cycles.
    The CFD simulations were only run for 2.5 cardiac cycles; the washout analysis was performed by looping through the last simulated cardiac cycle using the exported velocity fields in ParaView. We have clarified this in the method section and supplementary information.
12. - The discussion should focus on the significance of the findings. For example, how can we interpret a 5% reduction in flow stasis? Is it meaningful when it comes to the evaluation of thrombosis potential?
    - The discussion does not include any comment about the statistical insignificance derived from unpaired comparisons suggesting high inter-patient variability.
    We have added a paragraph to the discussion on the remaining blood after one cardiac cycle compared to two and three cycles. It should be noted that after changing to nonparameteric statistical tests, there was also a significant difference on a group wide comparison in AF patients before and after LAAO.

The remaining blood fraction after one cardiac cycle reduced on an individual level, but not on a group wide level and the patients with LAAO still had a significantly higher remaining blood fraction than the controls (both with LAA and after LAAO). After two and three cardiac cycles, the remaining blood in AF patients reduced significantly after LAAO compared to before also on a group wide level, with no significant difference to controls before LAAO. The increased stasis during the first cardiac cycle is probably caused by the generally enlarged atrial volume in the AF group, leading to

slower velocities in the LA. The blood remaining in the LA longer is mainly located in the LAA, and therefore reduced after LAAO.

13. - The authors state that LAAO reduces stasis, but the results show that some patients have higher stasis after LAAO compared with controls.
    - The results show that for some patients, LAAO does not yield favorable results in terms of improvement of hemodynamics. So these patients should not receive LAAO?

    *Our research shows that some patients still have high stasis even after LAAO. However, this was an in silico study on a relatively small group of patients, therefore, more research is needed to answer this question. We added to the discussion that '*More research is needed to examine if this is also the case during active fibrillation and if these patients have fewer benefits from LAAO.'
14. - The authors mention that LAAO could be considered for paroxysmal AF patients but they do neither evaluate nor discuss any clinical endpoints such as stroke reduction or thrombus formation. This is a huge drawback and needs to be addressed for the completion of the study. This makes this statement overreaching.

    The authors do not assess thrombus risk directly and therefore their statement that LAAO reduces stroke risk is far fetched.

    We have clarified the aim, so that it says 'evaluate how LAAO impacts the atrial hemodynamics of paroxysmal AF patients during sinus rhythm' and removed the statements on future clinical implications from the conclusions and the abstract.
15. - The limitations section should include that the occlusion was idealized while in reality devices may not have been placed in a manner that would be similar to how the authors assumed it to be.

    We added this concern to the limitations section.

Dear Dr Ebbers,

Re: JP-RP-2025-288587R1 "Virtual Left Atrial Appendage Occlusion Reduces Stasis in Paroxysmal Atrial Fibrillation Patients during Sinus Rhythm" by Sophia BÃ¤ck, Jonas Lantz, Iulia Skoda, Lars O Karlsson, Anders Persson, Carl-Johan Carlhäll, and Tino Ebbers

Thank you for submitting your manuscript to The Journal of Physiology. It has been assessed by a Reviewing Editor and by 2 expert referees and we are pleased to tell you that it is acceptable for publication following satisfactory revision.

The referee reports are copied at the end of this email.

Please address all the points raised and incorporate all requested revisions or explain in your Response to Referees why a change has not been made. We hope you will find the comments helpful and that you will be able to return your revised manuscript within 4 weeks. If you require longer than this, please contact journal staff: jp@physoc.org.

Your revised manuscript should be submitted online using the link in your Author Tasks Link Not Available. This link is accessible via your account as Corresponding Author; it is not available to your co-authors. If this presents a problem, please contact journal staff (jp@physoc.org). Image files from the previous version are retained on the system. Please ensure you replace or remove any files that are being revised.

If you do not wish to submit a revised version of your manuscript, you must inform our journal staff (jp@physoc.org) or reply to this email to request withdrawal. Please note that a manuscript must be formally withdrawn from the peer review process at one journal before it may be submitted to another journal.

TRANSPARENT PEER REVIEW POLICY: To improve the transparency of its peer review process The Journal of Physiology publishes online, as supporting information, the peer review history of all articles accepted for publication. Readers will have access to decision letters, including Editors' comments and referee reports, for each version of the manuscript, as well as any author responses to peer review comments. Referees can decide whether or not they wish to be named on the peer review history document.

ABSTRACT FIGURES: Authors are expected to use The Journal's premium BioRender account to create/redraw their Abstract Figures. Information on how to access this account is here:
https://physoc.onlinelibrary.wiley.com/journal/14697793/biorender-access.

This will enable Authors to create and download high-resolution figures. If authors have used the free BioRender service, they can use the instructions provided in the link above to download a high-resolution version suitable for publication.
The link provided should only be used for the purposes of this submission. Authors will be charged for figures created on this account if they are not related to this manuscript submission.

LANGUAGE EDITING AND SUPPORT FOR PUBLICATION: If you would like help with English language editing, or other article preparation support, Wiley Editing Services offers expert help, including English Language Editing, as well as translation, manuscript formatting, and figure formatting at www.wileyauthors.com/eeo/preparation. You can also find resources for Preparing Your Article for general guidance about writing and preparing your manuscript at www.wileyauthors.com/eeo/prepresources.

RIGOUR AND REPRODUCIBILITY: We recommend authors consult The Journal's Rigour and Reproducibility requirements to ensure that their manuscript is suitable for publication: https://physoc.onlinelibrary.wiley.com/pb-assets/hub-assets/physoc/documents/TJP-Rigour-and-Reproducibility-Requirements.pdf

REVISION CHECKLIST:
Check that your Methods section conforms to journal policy: https://jp.msubmit.net/cgi-bin/main.plex?form_type=display_requirements#methods

Check that data presented conforms to the statistics policy: https://jp.msubmit.net/cgi-bin/main.plex?form_type=display_requirements#statistics

Upload a full Response to Referees file. To create your 'Response to Referees' copy all the reports, including any comments from the Senior and Reviewing Editors, into a Microsoft Word, or similar, file and respond to each point, using font or background colour to distinguish comments and responses and upload as the required file type.

Please upload two versions of your manuscript text: one with all relevant changes highlighted and one clean version with no changes tracked. The manuscript file should include all tables and figure legends, but each figure/graph should be uploaded as separate, high-resolution files. The journal is now integrated with Wiley's Image Checking service. For further details, see: https://www.wiley.com/en-us/network/publishing/research-publishing/trending-stories/upholding-image-integrity-wileys-image-screening-service

You may also upload:
- 'Potential Cover Art' for consideration as the issue's cover image
- Appropriate Supporting Information (Video, audio or data set: see https://jp.msubmit.net/cgi-bin/main.plex?form_type=display_requirements#supp).

We look forward to receiving your revised submission.

If you have any queries, please reply to this email and we will be pleased to advise.

Yours sincerely,

Bjorn Knollmann
Senior Editor
The Journal of Physiology

REQUIRED ITEMS

(1) Your paper contains Supporting Information of a type that we no longer publish. Any information essential to an understanding of the paper must be included as part of the main manuscript and figures. The only Supporting Information that we publish are video and audio, 3D structures, program codes and large data files. Your revised paper will be returned to you if it does not adhere to our Supporting Information Guidelines.

Your supplementary excel data file is OK as supporting information, but the other file of supplementary material (simulation set-up) should really be included in the main article. You can include this as an Appendix at the end of the article file if it would interrupt the flow of the article otherwise.

(2) Please include a legend to accompany your Abstract Figure. This should be included in your article (Word) file, along with the other figure legends.

EDITOR COMMENTS

Reviewing Editor:

Your manuscript was assessed again and the expert referees highlight that most of their concerns have been addressed sufficiently. A few points remain to be addressed (see below).

The supplement listing many of the simulation parameters is useful (so should be included in the main article). As with complex simulation tools it is almost impossible to provide a comprehensive list simulation parameters, reproducibility would be strengthened by providing at least an example of an executable setup (mesh + simulation file).

Senior Editor:

I concur with the reviewing editor. There are a few minor deficiencies that remain to be addressed.

REFEREE COMMENTS

Referee #1:

Thank you, the manuscript has significantly improved in clarity. I have a few additional concerns:

1. Could you please clarify the need for performing the Washout Analysis to calculate the Remaining Blood % when the proposed Atrial Flow Component Analysis appears to accomplish the same goal, and possibly more?

2. The terms "blood fraction" and "Remaining Blood" are used interchangeably throughout the manuscript. Would you consider standardizing these terms to avoid any potential confusion?

3. The impact of other components (e.g., retained inflow, delayed outflow) discussed in the 5th paragraph still seems somewhat speculative. It might be useful to add a note in the limitations or future work section detailing how these components could be validated as predictors of thrombus formation-for example, through a study including patients with DRT.

4. Please address the citation errors (e.g., "Error! Reference source not found").

Referee #2:

The authors have addressed my concerns and comments. Congratulations on great work!

END OF COMMENTS

Comments from Editor

REQUIRED ITEMS

(1) Your paper contains Supporting Information of a type that we no longer publish. Any information essential to an understanding of the paper must be included as part of the main manuscript and figures. The only Supporting Information that we publish are video and audio, 3D structures, program codes and large data files. Your revised paper will be returned to you if it does not adhere to our Supporting Information Guidelines.

Your supplementary excel data file is OK as supporting information, but the other file of supplementary material (simulation set-up) should really be included in the main article. You can include this as an Appendix at the end of the article file if it would interrupt the flow of the article otherwise.

We added this information to the main article.

(2) Please include a legend to accompany your Abstract Figure. This should be included in your article (Word) file, along with the other figure legends.

We added a legend to our Abstract Figure.

The supplement listing many of the simulation parameters is useful (so should be included in the main article). As with complex simulation tools it is almost impossible to provide a comprehensive list simulation parameters, reproducibility would be strengthened by providing at least an example of an executable setup (mesh + simulation file).

As mentioned above, we moved the supplementary listing to the appendix of the main article.

We cannot upload the meshes for specific individuals, due to data privacy regulations from the ethical approval. However, the complete workflow from generating moving CFD meshes to setup the CFD simulations are handled through various scripts. These scripts are now available at this gitlab page: https://gitlab.liu.se/cim_public/atrialcfd_fluentexample. Using this workflow allows any user to use their own geometry/mesh for setting up the simulation.

We noticed a mistake in Table 1, where we swapped 'Systolic' and 'Diastolic'. We corrected this in the revised manuscript.

Reviewer 1

1. Could you please clarify the need for performing the Washout Analysis to calculate the Remaining Blood % when the proposed Atrial Flow Component Analysis appears to accomplish the same goal, and possibly more?

We have clarified the difference between the washout analysis and flow component analysis in the third paragraph of the discussion and added to the limitations that there is no established parameter to estimate stasis in the left atrium.

2. The terms "blood fraction" and "Remaining Blood" are used interchangeably throughout the manuscript. Would you consider standardizing these terms to avoid any potential confusion?

We have clarified this by rephrasing to 'remaining blood fraction'.

3. The impact of other components (e.g., retained inflow, delayed outflow) discussed in the 5th paragraph still seems somewhat speculative. It might be useful to add a note in the limitations or future work section detailing how these components could be validated as predictors of thrombus formation-for example, through a study including patients with DRT.

We added this to the limitations.

4. Please address the citation errors (e.g., "Error! Reference source not found").

We have addressed the citation errors.

Dear Dr Ebbers,

Re: JP-RP-2025-288587R2 "Virtual Left Atrial Appendage Occlusion Reduces Stasis in Paroxysmal Atrial Fibrillation Patients during Sinus Rhythm" by Sophia BÃ¤ck, Jonas Lantz, Iulia Skoda, Lars O Karlsson, Anders Persson, Carl-Johan Carlhäll, and Tino Ebbers

Thank you for submitting your manuscript to The Journal of Physiology. It has been assessed by a Reviewing Editor and by 1 expert referees and we are pleased to tell you that it is acceptable for publication following satisfactory revision.

The referee reports are copied at the end of this email.

Please address all the points raised and incorporate all requested revisions or explain in your Response to Referees why a change has not been made. We hope you will find the comments helpful and that you will be able to return your revised manuscript within 4 weeks. If you require longer than this, please contact journal staff: jp@physoc.org.

Your revised manuscript should be submitted online using the link in your Author Tasks Link Not Available. This link is accessible via your account as Corresponding Author; it is not available to your co-authors. If this presents a problem, please contact journal staff (jp@physoc.org). Image files from the previous version are retained on the system. Please ensure you replace or remove any files that are being revised.

If you do not wish to submit a revised version of your manuscript, you must inform our journal staff (jp@physoc.org) or reply to this email to request withdrawal. Please note that a manuscript must be formally withdrawn from the peer review process at one journal before it may be submitted to another journal.

TRANSPARENT PEER REVIEW POLICY: To improve the transparency of its peer review process The Journal of Physiology publishes online, as supporting information, the peer review history of all articles accepted for publication. Readers will have access to decision letters, including Editors' comments and referee reports, for each version of the manuscript, as well as any author responses to peer review comments. Referees can decide whether or not they wish to be named on the peer review history document.

ABSTRACT FIGURES: Authors are expected to use The Journal's premium BioRender account to create/redraw their Abstract Figures. Information on how to access this account is here: https://physoc.onlinelibrary.wiley.com/journal/14697793/biorender-access.

This will enable Authors to create and download high-resolution figures. If authors have used the free BioRender service, they can use the instructions provided in the link above to download a high-resolution version suitable for publication.
The link provided should only be used for the purposes of this submission. Authors will be charged for figures created on this account if they are not related to this manuscript submission.

LANGUAGE EDITING AND SUPPORT FOR PUBLICATION: If you would like help with English language editing, or other article preparation support, Wiley Editing Services offers expert help, including English Language Editing, as well as translation, manuscript formatting, and figure formatting at www.wileyauthors.com/eeo/preparation. You can also find resources for Preparing Your Article for general guidance about writing and preparing your manuscript at www.wileyauthors.com/eeo/prepresources.

RIGOUR AND REPRODUCIBILITY: We recommend authors consult The Journal's Rigour and Reproducibility requirements to ensure that their manuscript is suitable for publication: https://physoc.onlinelibrary.wiley.com/pb-assets/hub-assets/physoc/documents/TJP-Rigour-and-Reproducibility-Requirements.pdf

REVISION CHECKLIST:
Check that your Methods section conforms to journal policy: https://jp.msubmit.net/cgi-bin/main.plex?form_type=display_requirements#methods

Check that data presented conforms to the statistics policy: https://jp.msubmit.net/cgi-bin/main.plex?form_type=display_requirements#statistics

Upload a full Response to Referees file. To create your 'Response to Referees' copy all the reports, including any comments from the Senior and Reviewing Editors, into a Microsoft Word, or similar, file and respond to each point, using font or background colour to distinguish comments and responses and upload as the required file type.

Please upload two versions of your manuscript text: one with all relevant changes highlighted and one clean version with no changes tracked. The manuscript file should include all tables and figure legends, but each figure/graph should be uploaded as separate, high-resolution files. The journal is now integrated with Wiley's Image Checking service. For further details, see: https://www.wiley.com/en-us/network/publishing/research-publishing/trending-stories/upholding-image-integrity-wileys-image-screening-service

You may also upload:
- 'Potential Cover Art' for consideration as the issue's cover image
- Appropriate Supporting Information (Video, audio or data set: see https://jp.msubmit.net/cgi-bin/main.plex?form_type=display_requirements#supp).

We look forward to receiving your revised submission.

If you have any queries, please reply to this email and we will be pleased to advise.

Yours sincerely,

Bjorn Knollmann
Senior Editor
The Journal of Physiology

EDITOR COMMENTS

Reviewing Editor:

All issues are resolved from my point of view now. I agree with the referee that the title could be improved further.

Senior Editor:

I agree with the reviewer. The manuscript is acceptable but the title as written is not. Please edit and provide a more informative title that describes the main novelty of your modeling study.

REFEREE COMMENTS

Referee #1:

Thank you again for addressing my concerns, and congratulations on your research.

Regarding the title, you've clearly indicated the "virtual" nature of the occlusion, which is helpful. However, the core finding-that LAA occlusion reduces stasis-is a well-established principle. The main innovation of your work appears to be the application and perhaps specific advancements within your computational fluid dynamics (CFD) approach, potentially including your use of Flow Component Analysis. Therefore, I would consider revising the title to highlight these specific methodological contributions.

Otherwise, I have no further comments. And thanks for your patience.

END OF COMMENTS

Reviewer 1

Thank you again for addressing my concerns, and congratulations on your research.

Regarding the title, you've clearly indicated the "virtual" nature of the occlusion, which is helpful. However, the core finding-that LAA occlusion reduces stasis-is a well-established principle. The main innovation of your work appears to be the application and perhaps specific advancements within your computational fluid dynamics (CFD) approach, potentially including your use of Flow Component Analysis. Therefore, I would consider revising the title to highlight these specific methodological contributions.

Otherwise, I have no further comments. And thanks for your patience.

Thank you for your comment. Following your suggestion, we decided to change the title to:

Virtual Left Atrial Appendage Occlusion in Paroxysmal Atrial Fibrillation during sinus rhythm predicts variable reductions in Blood Stasis

Dear Professor Ebbers,

Re: JP-RP-2025-288587R3 "Virtual Left Atrial Appendage Occlusion in Paroxysmal Atrial Fibrillation during Sinus Rhythm predicts Variable Reductions in Blood Stasis" by Sophia Bäck, Jonas Lantz, Iulia Skoda, Lars O Karlsson, Anders Persson, Carl-Johan Carlhäll, and Tino Ebbers

We are pleased to tell you that your paper has been accepted for publication in The Journal of Physiology.

The last Word (or similar) version of the manuscript provided will be used by the Production Editor to prepare your proof. When this is ready you will receive an email containing a link to Wiley's Online Proofing System. The proof should be thoroughly checked and corrected as promptly as possible.

Authors should note that it is too late at this point to offer corrections prior to proofreading. Major corrections at proof stage, such as changes to figures, will be referred to the Editors for approval before they can be incorporated. Only minor changes, such as to style and consistency, should be made at proof stage. Changes that need to be made after proof stage will usually require a formal correction notice.

All queries at proof stage should be sent to: TJP@wiley.com.

Following copyediting and typesetting the accepted version will be published online.

Yours sincerely,

Bjorn Knollmann
Senior Editor
The Journal of Physiology

IMPORTANT POINTS TO NOTE FOLLOWING YOUR PAPER'S ACCEPTANCE:

If you would like to receive our 'Research Roundup', a monthly newsletter highlighting the cutting-edge research published in The Physiological Society's family of journals (The Journal of Physiology, Experimental Physiology, Physiological Reports, The Journal of Nutritional Physiology and The Journal of Precision Medicine: Health and Disease), please click this link, fill in your name and email address and select 'Research Roundup':
https://www.physoc.org/journals-and-media/membernews

• TRANSPARENT PEER REVIEW POLICY: To improve the transparency of its peer review process, The Journal of Physiology publishes online as supporting information the peer review history of all articles accepted for publication. Readers will have access to decision letters, including Editors' comments and referee reports, for each version of the manuscript as well as any author responses to peer review comments. Referees can decide whether or not they wish to be named on the peer review history document.

• You can help your research get the attention it deserves! Check out Wiley's free Promotion Guide for best-practice recommendations for promoting your work at: www.wileyauthors.com/eeo/guide. You can learn more about Wiley Editing Services which offers professional video, design, and writing services to create shareable video abstracts, infographics, conference posters, lay summaries, and research news stories for your research at: www.wileyauthors.com/eeo/promotion.

• IMPORTANT NOTICE ABOUT OPEN ACCESS: To assist authors whose funding agencies mandate public access to published research findings sooner than 12 months after publication, The Journal of Physiology allows authors to pay an Open Access (OA) fee to have their papers made freely available immediately on publication.

The Corresponding Author will receive an email from Wiley with details on how to register or log-in to Wiley Authors Services where you will be able to place an order.

• You can check if your funder or institution has a Wiley Open Access Account here: https://authorservices.wiley.com/author-resources/Journal-Authors/licensing-and-open-access/open-access/author-compliance-tool.html.

EDITOR COMMENTS

Reviewing Editor:

Title has been improved.

Senior Editor:

The manuscript is now acceptable. Thank you for your excellent contribution to the Journal!
